# Multifunctional Gelatin/Chitosan Electrospun Wound Dressing Dopped with *Undaria pinnatifida* Phlorotannin-Enriched Extract for Skin Regeneration

**DOI:** 10.3390/pharmaceutics13122152

**Published:** 2021-12-14

**Authors:** Carolina A. M. Ferreira, Adriana P. Januário, Rafael Félix, Nuno Alves, Marco F. L. Lemos, Juliana R. Dias

**Affiliations:** 1 CDRSP—Centre for Rapid and Sustainable Product Development, Instituto Politécnico de Leiria, 2030-028 Marinha Grande, Portugal; carolina.ferreira@ipleiria.pt (C.A.M.F.); nuno.alves@ipleiria.pt (N.A.); 2 MARE—Marine and Environmental Sciences Centre, ESTM, Instituto Politécnico de Leiria, 2050-641 Peniche, Portugal; adriana.p.januario@ipleiria.pt (A.P.J.); rafael.felix@ipleiria.pt (R.F.); marco.lemos@ipleiria.pt (M.F.L.L.)

**Keywords:** electrospun wound dressing, multifunctional structures, wound healing, chitosan/gelatin/phlorotannin-enriched extract, chronic wounds

## Abstract

The similarities of electrospun fibers with the skin extracellular matrix (ECM) make them promising structures for advanced wound dressings. Moreover, infection and resistance in wounds are a major health concern that may be reduced with antibacterial wound dressings. In this work, a multifunctional wound dressing was developed based on gelatin/chitosan hybrid fibers dopped with phlorotannin-enrich extract from the seaweed *Undaria pinnatifida*. The intrinsic electrospun structure properties combined with the antimicrobial and anti-inflammatory properties of phlorotannin-enrich extract will enhance the wound healing process. Electrospun meshes were produced by incorporating 1 or 2 wt% of extract, and the structure without extract was used as a control. Physico-chemical, mechanical, and biological properties were evaluated for all conditions. Results demonstrated that all developed samples presented a homogenous fiber deposition with the average diameters closer to the native ECM fibrils, and high porosities (~90%) that will be crucial to control the wound moist environment. According to the tensile test assays, the incorporation of phlorotannin-enriched extract enhances the elastic performance of the samples. Additionally, the extract incorporation made the structure stable over time since its in vitro degradation rates decreased under enzymatic medium. Extract release profile demonstrated a longstanding delivery (up to 160 days), reaching a maximum value of ~98% over time. Moreover, the preliminary antimicrobial results confirm the mesh’s antimicrobial activity against *Pseudomonas aeruginosa* and *Staphylococcus aureus*. In terms of biological characterization, no condition presented cytotoxicity effects on hDNF cells, allowing their adhesion and proliferation over 14 days, except the condition of 2 wt% after 7 days. Overall, the electrospun structure comprising phlorotannins-enriched extract is a promising bioactive structure with potential to be used as a drug delivery system for skin regeneration by reducing the bacterial infection in the wound bed.

## 1. Introduction

Skin is the major organ of the human body comprising vital functions namely the protection of internal tissues against external hazards [1]. Accordingly, when this barrier is compromised either by physical, chemical, or thermal injuries, it leads to wound development and consequent risks [2]. Currently, wounds are a burden for health care systems, presenting several typologies, ranging from acute surgical wounds, traumatic wounds, burn wounds, or chronic wounds (long-period healing), which require different treatments and multidisciplinary teams in health care facilities [2,3]. Associated with some of those wounds type, are underlying conditions such as (i) elderly population, (ii) diabetes (diabetic foot ulcer), and (iii) obesity, factors that continue to rise due to the increase of life expectancy [4]. The aforementioned debility conditions delay the wound healing process and may bring other complications such as bacterial infections [5]. In fact, when damage occurs, the wound is immediately contaminated with microorganisms that are part of the skin microflora. However, generally, healthy patients with acute wounds display a set of self-mechanisms to avoid the infection and promote self-healing [5]. On the other hand, burn and chronic wounds are prone to infection by bacteria such as *Staphylococcus aureus* and *Pseudomonas aeruginosa*, owing to the lack of protective barrier provided by the skin, weakness of host immune system, prolonged hospital stays, invasive diagnostic, and inefficient therapeutic procedures, which associated with the increasing resistance of bacteria to applied antibiotics delays the wound healing process [5,6,7].

In fact, a study of USA Medicare beneficiaries identified that 8.2 million people had wounds with or without infection, in 2018 [8], and between 25% and 40% of hospitals beds are occupied by patients with wounds [9]. These numbers reveal that available commercial products have low efficacy, while having high costs to the health care system [6]. To overcome these aforementioned drawbacks, it is crucial to improve the healing process through the development of advanced wound dressings which would greatly benefit from mimicking the biomechanical properties of the skin’s ECM. Based on this, remarkable progress has recently been made to develop advanced wound dressings through skin tissue engineering. Effectively, it is expected that the wound care market reaches US$27.8 billion by 2026, from which advanced wound care products represent the largest segment [10,11].

Electrospinning is a promising technique to produce advanced wound dressings based on electrostatic forces of a polymeric solution. When it is exposed to a high voltage field, and the threshold value of solution’s surface tension is exceeded, it forms a jet toward the collector. During this path the solvent gradually evaporates and the non-woven fibers are deposited [12]. This technique has been consolidated since it has the ability to produce randomly deposited fibers from sub-micron to nanoscale, mimicking the native skin ECM, which presents fibers with diameters from 10 to 300 nm [12,13]. Furthermore, it produces structures with (i) high porosity (60–90%) and interconnectivity to allow the passage of oxygen, nutrients, and control fluids loss [14], and (ii) high surface areas [15] promoting cell attachment [16], in an easy and fast way [17,18,19,20]. Another advantage of electrospun fibers, is the possibility to tailor the structure properties by ranging the parameters, namely: solution parameters (e.g., solvent, polymer, and concentration), ambient parameters (e.g., temperature and relative humidity), and processing parameters (e.g., nozzle, tip-to- collector distance, the voltage applied, and flow rate) [21,22]. Additionally, it is considered a versatile technique since it enables the use of several polymers, like natural polymers, namely gelatin and chitosan, that are both biocompatible, nontoxic, and biodegradable. Chitosan has hemostatic properties, reduces scar formation, and promotes a good tissue reepithelization [14], alongside with having reported antimicrobial activity [23,24]. Gelatin presents arginine-glycine-aspartic acid (RGD) sequences that promote cell proliferation [25], thus being commonly applied in wound healing [12,26,27]. Thus, over the past few years, electrospinning has been consolidated as a promising technique as a drug delivery system (DDSs) due to the electrospun meshes high surface-to-volume area and porosity that provide a large contact area improving drug dissolution [22]. Moreover, the versatility of electrospinning applications enables its use for site-specific delivery of drugs to the body [28], which can have oral, topical, transdermal, and transmucosal administration [22]. Similarly, it is possible to encapsulate different drugs and/or biomolecules, through core/shell (co-axial), emulsion, or simultaneous electrospinning approaches [15,21,29] and thus improving therapeutic efficacy, drug bioavailability, and reducing the toxicity by delivering them at a controlled rate [15,22].

In recent years, bacterial antibiotic resistance led to the urge to study and bioprospecting novel natural compounds, which possess characteristics such as good therapeutic anti-inflammatory, antimicrobial, and antioxidant potentials, as well as lower toxicity, side effects, and cost [30]. The phlorotannins of brown seaweeds, such as the invasive *Undaria pinnatifida* (Harvey Suringar 1873), have several bioactivities, namely antimicrobial activity against wound’s infection bacteria such as *S. aureus* and *P. aeruginosa* [31,32,33] making it a potential natural drug. Indeed, Gram-positive *S. aureus* is responsible for the first stage of the infection, while, the infections caused by the Gram-negatives such as *Escherichia coli* and *P. aeruginosa* occurs when the wound is in the advanced phase [34,35]. The mechanisms of action of phlorotannins against the above-mentioned bacteria remains unidentified due to complex structure that phlorotannins can present. Nevertheless, it is pointed out that antimicrobial activity is related to the phlorotannins hydroxyl group scavenging activity, which allows the interaction with bacterial cell membrane causing its disruption. Other associated mechanism is by causing harmful effects on bacterial proteins, by reacting with the amine groups [32,36]. Another described activity of those phlorotannins compounds is the antioxidant activity, which is important to combat the reactive oxygen species (ROS) produced by inflammatory cells (e.g., macrophages) in wound repair [35,37]. Excessive concentrations of ROS inhibits wound healing, causing chronic inflammation and cell death, and is frequently reported in chronic and burn wounds [35]. Those compounds, also have reported anti-inflammatory activities, mainly against nitric oxide (NO), which in excessive concentrations, is responsible for continuous inflammation causing cellular death and avoids reepithelization [33,37,38]. The encapsulation of phlorotannins into electrospun fibers overcome some limitations at the wound site, improving their stability, release kinetics increasing its performance and effectiveness. At the same time, phlorotannins may reduce the penetration of enzymes such as collagenase, elastase, and matrix metalloproteinases (MPP) which are responsible for ECM degradation and reepithelization inhibition in chronic wounds [39,40].

Other studies in the tissue engineering field already incorporate polyphenols (the group of phlorotannins) like tannic acid, curcumin, thymol [41] in hydrogels, electrospun fibers, or scaffolds [4,42,43,44]. In these studies they conjugated polyphenols with synthetic polymers such as polycaprolactone (PCL), poly (vinyl alcohol) (PVA), or (polyethylene oxide) PEO, due to polyphenols’ biological activities as well as their characteristics that allow enhancing mechanical properties via chemical bonding [39,40].

To the best of knowledge, this work describes for the first time the development of an electrospun skin substitute based on gelatin and chitosan fibers that, not only, mimic the skin ECM, but also potentially preventing infections by acting as a drug delivery system of a phlorotannins-enriched extract from *Undaria pinnatifida*.

## 2. Materials and Methods

### 2.1. Materials

Gelatin powder of pig skin (type A, 300 bloom, 60 mesh) was kindly supplied by Italgelatine (Santa vittoria d’Alba, Italy), and chitosan (molecular weight: 100,000–300,000; 9012-76-4, ≥75% deacetylation) was acquired from ACROS Organics™ (Geel, Belgium). Acetic acid (AA; 64-19-7, ≥99%) glacial was purchased from Scharlau (Barcelona, Spain). Triethylamine (TEA; 121-44-8, ≥99%) was from Sigma Aldrich (St. Louis, MO, USA), 1,4-butanediol diglycidyl ether (BDDGE, 2425-79-8, ≥96%) from Alfa Aesar (Haverhill, MA, USA), glutaraldehyde (GA; 111-30-8; Grade I; 25% in H_2_O) and ethanol absolute (64-17-5; ≥ 99%) were also purchased from Sigma Aldrich (St. Louis, MO, USA). All materials used were of analytical grade and used without any further purification.

### 2.2. Phlorotannin-Enriched Extract Preparation

Phlorotannin-enriched extract used in this study was obtained using a sequential extraction as described by Ferreira et al. [33] using the invasive seaweed *Undaria pinnatifida*. Briefly, the obtained ethanolic extract from the sequential extraction was semi-purified through liquid–liquid extraction procedures, using hexane, which originates the ethanol wash fraction used in this work. Prior to extract incorporation, the bioactive properties such as antimicrobial, antioxidant, and anti-inflammatory were assessed as a proof-of-concept to use in wound healing, as reported in Ferreira et al. [33] work.

### 2.3. Electrospun Meshes Preparation

To produce electrospun fibers, the initial polymeric solution was prepared based on Pezeshki-Modaress and co-workers [45]. After fibers optimization the selected solution parameters were 14 wt% of gelatin and 3.6 wt% of chitosan dissolved in AA at 70% (*v*/*v*), and 2% (*v*/*v*) of TEA was added to increase the solution conductivity. The solution was stirred overnight at 37 °C to obtain a homogeneous solution. Then, phlorotannins-enriched extract (1 or 2 wt%) was added to the previous solution and stirred overnight, right after electrospun fibers production 4% (*v*/*v*) of BDDGE was added, as described by Dias et al. [12], to improve the structural stability in aqueous media. The amount of extract incorporated considered the bioactivities previously tested [33], to ensure the electrospun meshes biofunctionality. Additionally, concentrations higher than 2 wt% were experimentally tested, however, no fibers were produced. Electrospun fibers were produced using a home-made apparatus with the optimized parameters (20 kV, 12 cm, and 0.2 mL·h^−1^). Three different samples were produced: (i) gelatin/chitosan, without extract (control meshes)–WOE; (ii) gelatin/chitosan with 1 wt% of phlorotannin-enriched extract—WE1; and (iii) gelatin/chitosan with 2 wt% of phlorotannin-enriched extract—WE2.

### 2.4. Physicochemical Characterization

#### 2.4.1. Apparent Density and Porosity

The apparent density and porosity of electrospun meshes were calculated using the Equations (1) and (2).

(1) $$\mathrm{Apparent}\;\mathrm{density}\;\left(g\cdot {\mathrm{cm}}^{-3}\right)=\frac{\mathrm{mesh}\;\mathrm{mass}\;\left(g\right)}{\mathrm{mesh}\;\mathrm{thickness}\;\left(\mathrm{cm}\right)\cdot \mathrm{mesh}\;\mathrm{area}\;\left({\mathrm{cm}}^{2}\right)}$$

(2) $$\mathrm{Mesh}\;\mathrm{porosity}\;\left(\%\right)=\left(1-\frac{\mathrm{mesh}\;\mathrm{apparent}\;\mathrm{density}\;\left(g\cdot {\mathrm{cm}}^{-3}\right)}{\mathrm{Bulk}\;\mathrm{density}\;\mathrm{of}\;\mathrm{gelatin}+\mathrm{chitosan}\;\left(g\cdot {\mathrm{cm}}^{-3}\right)}\right)\times 100$$

For mesh porosity, bulk densities were calculated as in [46].

#### 2.4.2. Morphology and Fiber Diameter

Scanning electron microscopy (SEM) Vega3-LMU (TESCAN, Kohoutovice, Czech Republic) was used to evaluate the electrospun meshes morphology. The meshes were coated with gold/palladium (Au/Pd) thin film by sputtering (Quorum Technologies, Lewes, UK) before the examination. ImageJ (Fiji, version J1.46r., Würzburg, Germany) was used to estimate the fibers diameter distribution through the average of fifty measurements per image of three independent meshes.

#### 2.4.3. Chemical Characterization

To analyze the interaction between the compounds used to produce the electrospun meshes and detect possible structural changes, Fourier transform infrared spectroscopy with attenuated total reflectance (FTIR-ATR) was used. The analysis was carried out using an Alpha-P, FTIR-ATR spectrometer (Bruker, Billerica, MA, USA), in the range of 4000–400 cm^−1^, at a 4 cm^−1^ of resolution with 64 scans at room temperature.

#### 2.4.4. Dissolvability and Water Uptake

To quantify the dissolvability, five samples of each condition (WOE, WE1, and WE2) were dried for 24 h before weight determination, followed by immersion in distilled water. After 24 h of incubation, the samples were removed from distilled water and the excess of water was removed and weighed again, and the swelling ratio was calculated according to the following equation:

$$\mathrm{Swelling}\;\mathrm{degree}\;\left(\%\right)=\frac{\mathrm{Ww}-\mathrm{Wd}}{\mathrm{Wd}}\times 100$$

where Ww is the wet weight and Wd is the dry weight.

Then, the samples were dried at 37 °C for an additional 24-h period and weighed to evaluate their dissolvability.

$$\mathrm{Dissolvability}\;\left(\%\right)=\frac{W0-\mathrm{Wd}}{W0}\times 100$$

where W0 is the initial weight, before the experiment, and Wd is the dry weight.

#### 2.4.5. Water Vapor Permeability

The electrospun meshes water vapor permeability (WVP) was carried out following the ASTM E 96-00 standard test for water vapor transmission [47]. Briefly, the meshes were attached to the vials aperture, which were previously filled with 5 mL of phosphate-buffered saline (PBS) solution, having a vapor permeation area of 3.60 cm^2^. For this experiment, five vials were used, and each vial was weighed and kept at 32° C. After 24 h, the WVP was calculated from the weight changes according to the next equation:

$$\mathrm{Water}\;\mathrm{vapor}\;\mathrm{permeability}\;\left(\mathrm{WVP}\right)=\frac{\Delta W}{\mathrm{tA}}$$

where ΔW is water weight change (g), t is the time (h), and A is the test area (vials aperture area), in m^2^.

#### 2.4.6. Contact Angle

The contact angle of electrospun meshes was measured by a Theta Lite Tensiometer (Attension, Espoo, Finland) and the image analysis was performed using One Attension software, with Laplace approach. A droplet of deionized water (20 µL) was placed on the mesh surface and the measurement started immediately. The assay was recorded for 12 s at a speed of 15 frames per second. Curve fitting was applied to measure the contact angle for a theoretical meridian drop profile between the baseline and the tangent to the drop boundary. The contact angle of each electrospun structure was measured immediately after the drop touched the mesh surface (time 0 s) and after 5 s. Three replicates were performed per sample, with mean and standard deviation (± SD) values reported.

### 2.5. Mechanical Properties

The mechanical properties of all conditions (WOE, WE1, and WE2) were measured using the tensile test in the texturometer TA.XT Plus model (Stable Micro System SMD, Surrey, UK) with a 5N load cell. The tensile tests were performed with the samples in a wet state, a gauge length of 10 mm, and a test speed of 1 mm s^−1^. At least five individual samples from each condition were tested and measurements were reported as mean ± SD.

### 2.6. Hydrolytic and Enzymatic Degradation

Hydrolytic and enzymatic degradations were performed to evaluate the performance of electrospun meshes, based on weight changes over time. In hydrolytic degradation, the samples were placed in tubes filled with 5 mL of PBS (8 g NaCl, 0.2 KCl, 1.44 g Na_2_HPO_4_·12H_2_0, 0.2 g K_2_HPO_4_ in 1 L of distilled water, pH 7.4) and 0.02% (*w*/*v*) of sodium azide, to work as a bacteriostatic agent. On the other hand, in enzymatic degradation, the samples were immersed in 5 mL of PBS with 6.5 mg·L^−1^ of lysozyme from chicken egg white (Sigma-Aldrich, St. Louis, USA). The presence of this enzyme in the wound bed is an indicator of beginning of infection since their activity increases in infected wounds (4830 ± 1848 U·mL^−1^) when compared to non-infected wounds (376 ± 240 U·mL^−1^) [48]. Besides, it is responsible for the binding cleavage of some components of the bacterial cell wall as well as the degradation of chitosan and gelatin [49,50]. Based on this data, an intermediate concentration (6.5 mg·L^−1^) of this enzyme in serum was chosen to perform the assay. The samples were incubated in an orbital shaker incubator KS 4000i control (IKA, Staufen, Germany) at 37 °C with a constant stirring of 100 rpm for 28 days. In the enzymatic set, to ensure the lysozyme activity, the medium was changed twice a week. In the hydrolytic degradation the medium was changed only once a week. During the experiment, for the time points of 3, 7, 14, 21, and 28 days, the samples of both sets were collected, and their wet weight was recorded. After that, the samples were dried for 24 h at 37 °C, and in the end, samples’ dry weight was registered to calculate the mesh degradation.

### 2.7. Extract Delivery

The release of the two different concentrations of phlorotannins-enriched extract from meshes was carried out by UV-spectrophotometry SPECTROstarNano (BMG Labtech, Ortenberg, Germany) at a wavelength of 270 nm with phlorotannin-enriched extract as standard. The entire meshes were placed into 15 mL tube containing 5 mL of PBS at pH 6 and incubated in an orbital shaker at 37 °C until the complete release was achieved. When the skin suffers an injury and with the body’s response to the inflammatory phase, the pH tends to be acidic proximally to the neutral environment (~6) to promote faster healing [51]. At certain time points (30 min, 1, 2, 4, 6, 8, 24, 48, 72 h, and once a week until the end of the experiment), the UV absorbance values of release solutions were measured using the microplate reader, while the content was replaced for new PBS solution. The release was measured accumulatively using linear regression with a correlation of 0.99. Extract release was carried out with the mathematical model of Korsmeyer and Peppas [52], with a polymeric system equation:

$$\mathrm{Log}\;\left(\mathrm{Mt}/M\infty \right)=\mathrm{Log}\;K\;+\;n\;\mathrm{Log}\;t$$

where Mt/M∞ is a fraction of drug released at time t, K is the release rate constant, and n is the release exponent that characterizes the release mechanism and its dependency on the structure geometry and the physical mechanism of release [53].

The morphology of the electrospun structure was evaluated by SEM (previously described in Section 2.4.2) 24 h after the extract release, and 160 days after.

### 2.8. Antimicrobial Activity by Disc Diffusion Assay

Due to its prevalence in wounds, as aforementioned, the reference strains used were *S. aureus* (ATCC 25923) and *P. aeruginosa* (ATCC 27853). Similar to the work described by Ferreira et al. [33], the antimicrobial activity of meshes was performed according to the Clinical and Laboratory Standard Institute (CLSI, 2014) [54], reference documents M02 and M100-S25. In brief, *S. aureus* and *P. aeruginosa* were freshly overnight prepared in tryptone soya yeast extract agar (TSYEA, Sigma Aldrich, St. Louis, MO, USA) and nutrient agar (NA, Sigma Aldrich, St. Louis, MO, USA), respectively, at 35 °C. Afterward, colonies of bacteria were resuspended in 0.85% saline solution and turbidity adjusted to 0.5 McFarland (approximately 1.5 × 10^8^ CFU·mL^−1^). Then, the inoculum was spread with a swab on Muller-Hinton agar (MHA) plates. Four meshes (8.5 mm in diameter) were placed in the above MHA plate. As positive controls, disc containing ciprofloxacin were used (CIP, 5 μg; Liofilchem, Italy). After 18 h of incubation at 35 °C, the formation of a halo was evaluated. The experiment was carried out three times and halo measurements were performed using the software ImageJ. The normalized widths of the antimicrobial “halo” of each disc was calculated by applying the following equation [55]:

$$\mathrm{Normalized}\;\mathrm{widths}\;\mathrm{of}\;\mathrm{the}\;\mathrm{antimicrobial}\;halo\;(nw_{halo})=\frac{\frac{D_{iz}-d}{2}}{d}$$

where “*D_iz_*” is the diameter of the inhibition zone and the “*d*” is the disc diameter (8.5 mm).

### 2.9. In Vitro Studies

In vitro studies were performed using Human dermal neonatal fibroblasts (hDNF) isolated from the foreskin of healthy male newborns (ZenBio, Durham, UK) cultured, expanded, and maintained in Dulbecco’s modified eagle medium (DMEM) (Sigma Aldrich, St. Louis, MO, USA), supplemented with 10% *v*/*v* fetal bovine serum (FBS, Sigma Aldrich, St. Louis, MO, USA), 1% *v*/*v* of penicillin solution (Sigma Aldrich, St. Louis, MO, USA), and 1% *v*/*v* of amphotericin B solution (Sigma Aldrich, St. Louis, MO, USA). The medium was changed twice a week, and cells were incubated at 37 °C in a 5% CO_2_ humidified atmosphere incubator. When the cells reached 80–90% of confluence, they were detached using a trypsin solution (0.25% trypsin/0.05% ethylenediaminetetraacetic acid (EDTA, Sigma Aldrich, St. Louis, MO, USA)/0.1% glucose in PBS (pH 7.4). In vitro studies used cells from passages between 5 and 11.

#### 2.9.1. Cytotoxicity

The cytotoxicity of electrospun meshes was tested in accordance with the International Standard ISO 10993-5 [56], with direct (samples) and indirect (leachables) methods. Samples were prepared by cutting the meshes in a circular shape (8.5 mm diameter) and weighing them. Control meshes with gelatin and chitosan were previously sterilized under UV light (253.7 nm) for 15 min on each side. Previously to the cytotoxicity assay, samples were washed in ultrapure water and, for indirect contact, were incubated during 24 h in DMEM medium. Meanwhile, hDNF were seeded at a density of 2 × 10^4^ cells/well and incubated for 24 h at the conditions previously described. After this period, for the indirect contact set, the culture medium was replaced by the medium that was in contact with samples (leachables), while for direct contact, the culture medium was replaced by a fresh medium, and the samples were placed in contact with the cells. Positive controls (viable cells) were maintained by culturing cells with DMEM medium. Cells were further incubated for a period of 24 h, and after this time, the cells’ metabolic activity for each sample was evaluated by the colorimetric viability assay, using resazurin [12]. Briefly, after 24 h the culture medium was replaced by a mixture of 80% DMEM medium and 20% of resazurin solution (0.01 mg·mL^−1^ in PBS), and incubated at 37 °C for 2 h. After the incubation time, 300 µL per well were transferred to a black 96-well plate and make triplicates of 100 µL. Viability was measured using a microplate reader (FLUOstar Omega, BMG Labtech, Ortenberg, Germany), with excitation and emission wavelengths of 530 nm and 590 nm, respectively.

#### 2.9.2. Proliferation Assays

To guaranty the cell integration into the meshes and to avoid cell agglomeration on the well bottom, the seeding was performed with 10 µL of cells at a density of 1 × 10^4^ medium and incubated for 2 h. After that 500 μL of medium was added and cultured for 1, 7, and 14 days, replacing the medium twice a week. Afterwards, the cell proliferation was evaluated by resazurin assay, previously described, using electrospun meshes without cells seeded as a control [12,57]

To visualize the spread of hDNF cells into the meshes and evaluate their morphology, SEM analysis was performed (previously Section 2.4.2). For that, the cells were first washed with PBS, and afterward fixed for 30 min in 2.5 wt% glutaraldehyde and dehydrated with a successive graded ethanol series (40, 50, 70, 90, and 100%) for 15 min each.

### 2.10. Statistical Analysis

The results were expressed as mean ± standard deviation (SD). Statistical analysis (Levene’s and *t*-test) was carried out using SigmaPlot (version 11.0, Systat Software Inc, San Jose, CA, United States of America (USA)) with a 95% confidence level, for porosity, average fiber diameter, swelling degree, dissolvability procedures, and mechanical tests. In biological behavior, namely cytotoxicity, the data were checked for normality using Kolmogorov–Smirnov test, followed by t-test. In the proliferation assay, Shapiro–Wallis was used to ensure normality, and one-way analysis of variance (ANOVA) followed by Tukey test, equally for antimicrobial activity. A *p*-value less than 0.05 (*p* ≤ 0.05) was considered statistically significant.

## 3. Results and Discussion

### 3.1. Morphology and Fiber Diameter

**Figure 1:**
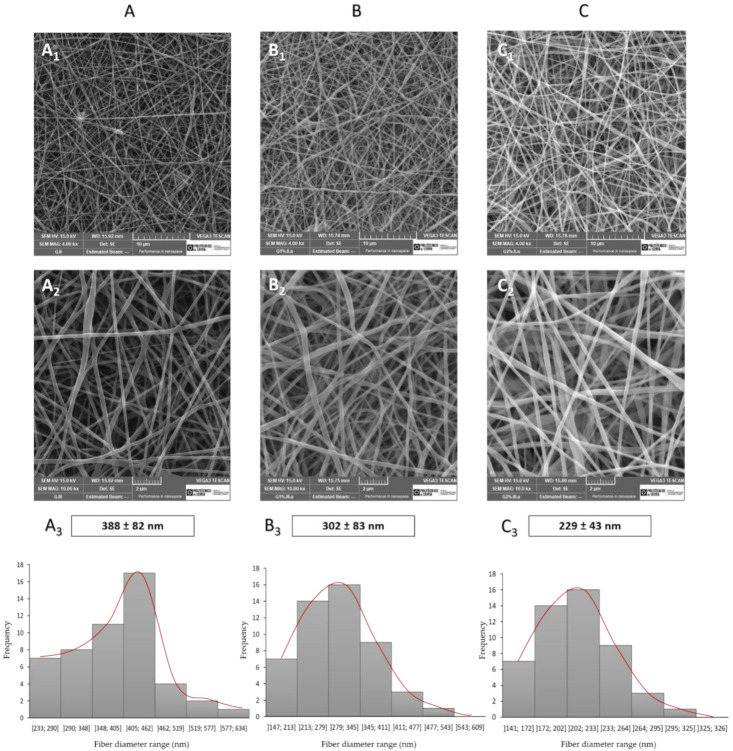
Scanning electron microscopy morphological assessment of electrospun meshes. (**A**) WOE electrospun meshes at (**A_1_**) 4000 × and (**A_2_**) 10,000 × of magnification and respective average fiber diameters with the distribution curve (**A_3_**); (**B**) WE1 electrospun meshes at (**B_1_**) 4000 × and (**B_2_**) 10,000 × of magnification and respective average fiber diameters with the distribution curve (B_3_); (**C**) WE2 electrospun meshes at 4000 × (**C_1_**) and 10,000 × of magnification (**C_2_**), and respective average fiber diameters with the distribution curve (**C_3_**).

Skin regeneration dressings should cover and protect the wound from external factors such as bacterial infections and keep the ideal moist environment, which is made possible with electrospun fibers due to their porosity. Therefore, electrospun dressings must achieve some requirements such as high porosity (60–90%) and interconnectivity to allow the passage of oxygen, nutrients, and cells [17]. The produced meshes morphology was evaluated by SEM at two different magnifications (Figure 1). The sample A represents gelatin/chitosan without extract (WOE), while B is the gelatin/chitosan sample with 1 wt% of phlorotannin-enriched extract (WE1), and C is gelatin/chitosan sample with 2 wt% of phlorotannin-enriched extract incorporated.

The meshes’ porosity is influenced by a range of parameters, such as fiber diameter and fiber density per area, which are a consequence of the production parameters selected [12]. The SEM images of all conditions show meshes with fibers randomly oriented and continuous without beads resulting in a homogenous structure. From the SEM images analysis, it was observed that WOE samples and WE1 meshes present similar fiber diameters, 388 ± 82 nm and 302 ± 83 nm, respectively, without significant differences (*p* > 0.05).

Nevertheless, by increasing extract concentration the fibers diameter decreases, as observed for WE2 meshes (229 ± 43 nm). In fact, those fibers had significant differences when compared with the WOE samples and WE1 (*p* ≤ 0.05) probably due to the chemical interaction between extract and polymers. These results are corroborated by the study of Lu et al. [26], where the gelatin/chitosan dressings crosslinked with tannic acid (TA), a polyphenol as phlorotannins, ranging in size from 100 to 240 µm, showed single fibers of gelatin or chitosan with greater size ranges and irregular distribution (90–280 µm). Thus, the diameters obtained fall within the diameter range of the skin ECM fibers, which exhibit diameters between 10 and 300 nm [21].

Fiber diameter has been described to have a significant influence on cell adhesion and proliferation since fibers with small diameters produce membranes with high surface area and increase the synthesis of collagen and proteoglycans from fibroblasts [24]. On the other hand, fibers with a small diameter decrease the porosity of the structure and may limit the ability of cell infiltration [58]. All conditions present porosity values over 85%, namely 85.93 ± 1.58% for WE2, 88. 63 ± 1.81% for WOE, and 89.22 ± 1.15% for WE1 meshes. These results can be correlated with the fibers’ diameters since reduced fiber diameter leads to a more compact meshes decreasing the porosity. Despite there are no statistically significant between all conditions, it is important to highlight that the range of fibers diameters obtained induce small pores that are able to protect the damaged tissue from pathogenic bacteria, avoiding their entry into the wound [59].

### 3.2. Physiochemical and Structural Characterization

**Figure 2:**
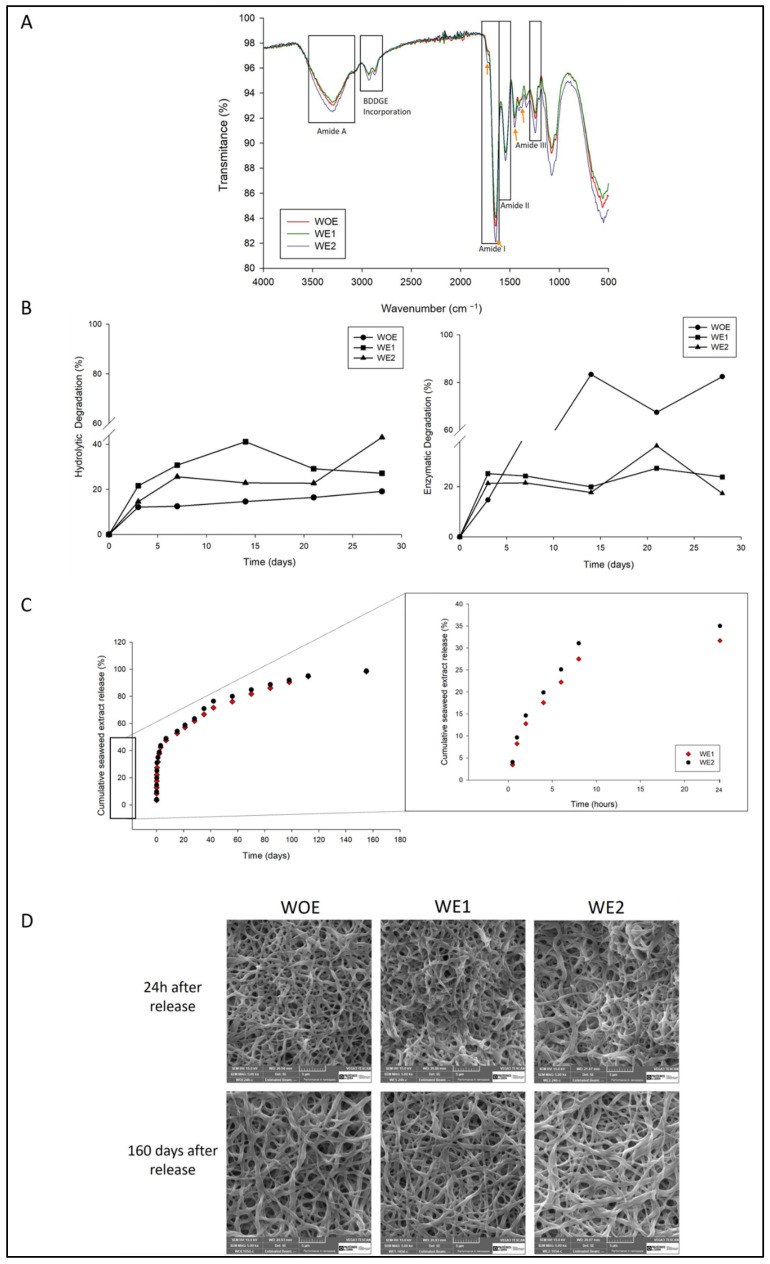
(**A**) FTIR spectra of WOE mesh (red line), WE1 mesh (green line) and WE2 mesh (blue line). The orange arrows correspond to the phlorotannins band at 1730 cm^−1^, 1612 cm^−1^,1482 cm^−1^, and 1382 cm^−1^. (**B**) Degradation kinetics over 28 days (n = 5), on left: hydrolytic degradation; on right: enzymatic degradation. (**C**) Release profile of phlorotannins-enriched extract from gelatin/chitosan electrospun meshes over time (WE1 and WE2) with meshes without extract as control (WOE) (n = 5), in days on left and the initial release in hours on right. (**D**) SEM images panel of WOE, W1E, and WE2 24 h after extract release, and after 165 days with a magnification of 5000 ×.

FTIR analysis was performed to evaluate the chemical composition of fibers and evaluate the interaction between gelatin, chitosan, BDDGE and the influence of phlorotannins-enriched extract, using gelatin-chitosan fibers as control. The obtained spectra are shown in Figure 2A. The FTIR spectrum of gelatin comprised several bands, at 1646 cm^−1^ which is consistent with the amide I band, due to the presence of carbonyl (C=O) group [60,61]. A prominent band in the region of 1545 cm^−1^ is notable and it is consistent with amide II with vibration of N-H groups and stretching vibrations of C–N groups. The band at the region of 1242–1444 cm^−1^ matching with amide III wavelengths, corresponds with the vibrations in plane of C–N and N–H groups of bound amide or vibrations of CH_2_ groups of glycine [12,29,60,61]. The main characteristics bands of chitosan are around 3353–3284 cm^−1^ attributed to O–H and N–H stretching vibrations of the functional group engaged in intramolecular hydrogen bonding between chitosan molecules, and in 1623.13, 1526.84, and 1425.7 cm^−1^ corresponding to the amide I, attributed to C-O stretching, N–H bending vibration of the amide II and, N–H and C–N vibrations of amide III, respectively [62]. The incorporation of BDDGE is also perceived at the spectrum, between the range of wavelengths of 2930–2890 cm^−1^ as a result of aliphatic moieties from this crosslinker [12]. Changes in amplitude and intensity of amide I and II bands produced by BDDGE were hardly noticeable suggesting a possible interaction through hydrogen bonds at this level to be negligible [63]. The spectra for samples containing phlorotannins have a narrow band at 1733 cm^−1^ that can be related to C=O stretching vibration of O-acetyl groups [33], in 1612 cm^−1^region, between 1463 cm^−1^ and 1482 cm^−1^, and 1375 cm^−1^ band which is slightly sifted to 1382 cm^−1^ (all indicated by an orange arrow) corresponding to the aromatic ring structure typical of these compounds, 3300 cm^−1^ for phenoxyl group and 1300 cm^−1^ for aromatic ether stretch [4]. According to the results, the chemical structure was not affected by the extract addition as well as with BDDGE. In addition, the increased intensity of some bands, related especially with amino and carbonyl, may evidence interaction between these groups, through electrostatic interactions (poly-anion/cation complexes by gelatin/chitosan) and also, hydrogen bonds (of O-H and N-H), leading to a miscible solution [60,61,64,65].

### 3.3. Water Uptake, Dissolvability, Water Permeability, and Contact Angle

Water absorption and water retention indicate the dressing’s ability to maintain an adequate wet environment in wound areas that is crucial to achieving faster wound healing [61]. Dry environments cause cell death, and leads to crust and scar formation, hence to form new tissue fibroblasts it will have to pass through that crust which generates greater energy consumption and delays healing [43,66,67]. The maintenance of the wound environment is achieved by exchanging gases through the porous structure and by the ability to absorb the exudate, which leads to bacteria proliferation and wound healing delay [43,66].

According to the results (Table 1), the extract incorporation increases the swelling degree when compared to the WOE samples, which may be due to the hydrophilic character of phlorotannins-enriched extract. Similar studies conducted by Kim and his colleagues demonstrated the same swelling behavior for fibers containing phlorotannins since extract incorporation improved the meshes’ water absorption [4].

To keep the electrospun meshes stable under a moist environment, the samples were crosslinked with BDDGE as aforementioned. To assess, indirectly, the crosslinking degree, the dissolvability assay was performed for 24 h, to evaluate the non-crosslinked material. The results show similar behavior between WOE meshes, and the WE2 meshes, with a dissolvability of 9.02 ± 0.67% and 9.05 ± 0.96%, respectively. There was a higher mass loss in WE1 meshes, 16.35 ± 0.52% after 24 h (*p* < 0.05). These results agree with the results obtained in the hydrolytic degradation. It is notorious that they are correlated with the fibers diameter and consequently with structures porosity, but also, probably due to the instability that 1 wt% of extract incorporation induces during the rearrangement of the molecules throughout the polymeric solution stretching. Moreover, the addition of BDDGE and phlorotannin-enriched extract decreases the solution pH, resulting in higher protonation that hampers the achievement of 100% of crosslink [12,63].

An ideal wound dressing must guarantee an adequate water vapor permeability (WVP) rate to ensure wound protection against an excessive dry or wet environment. The WVP rate was evaluated for each condition (see Table 1), the WOE meshes present a permeability of 1207.06 ± 14.97 g·m^−2^·day^−1^, while WE1 fibers have a slightly higher permeability of 1220.726 ± 12.06 g·m^−2^·day^−1^, and the WE2 meshes present a value of 1201.06 ± 6.00 g·m^−2^·day^−1^, however without statistical significance (*p* > 0.05) between them. The obtained results are similar to those of the study of Letha et al. [68] where polyurethane-gelatin, an electrospun fiber, displayed a WVP of 1172 g·m^−2^·day^−1^. The WVP rate is influenced by the wound type and if it produces higher or lower amounts of exudate, as well as by the fiber diameter and mesh porosity. The extract introduction does not seem to cause significant alterations, as occurred in similar work, where chitosan composites crosslinked with the polyphenol TA resulted in the reduction of WVP (increase the vapor barrier property) likely due to the decreasing the free -OH groups, creating a vapor diffusion path through the film [69]. Despite the value of WVP for normal skin is 204 g m^−2^ day^−1^, and for first-degree burn and injured skin between 279 and 5138 g m^−2^ day^−1^ [67,70], an ideal wound dressing should be close to 2000–2500 g m^−2^ day^−1^ to provide an adequate level of moisture without wound dehydration [70,71,72]. The obtained values are lower than the recommended range, however available products with good performance presents lower WVP values, such as the cases of Comfeel^®^, Dermiflex^®^, Tegaderm^®^, or OpSite^®^ with 285, 76, 491, and 792 g·m^−2^ day^−1^, respectively [73].

Wound dressings must have a hydrophilic character since hydrophobic surface materials are a vehicle for bacteria colonization and hydrophilicity contribute significantly to cell attachment, cell proliferation, and cell migration [4,66]. To evaluate the meshes water affinity, the water contact angle (WCA) methodology was performed (see the water contact angle vs. time profile of the electrospun meshes on Figure S1, Supplementary Materials). The WOE meshes showed a high hydrophilic profile since its contact angle is 0°. By increasing the amount of extract enriched with phlorotannins, the water absorption was delayed in the first seconds, being completely absorbed after 5 s. In fact, for the WE1 mesh, the WCA passes from 54.53°± 5.01 in the first 0.5 sec to 0° after 5 s. The same behavior was observed for WE2 samples, which have a WCA of 63.69° ± 4.47 in 0.5 s, and 5 s later have 0°. The addition of seaweed extract in the fibers allows maintaining the polar profile of the meshes, since the extract is expected to be hydrophilic due to phloroglucinol polymers which contain hydroxyl groups, and a higher number of hydroxyl groups increases the polar component of surface free energy resulting in increased hydrophilicity [4]. Additionally, water molecules take longer to penetrate the surface of the mesh, probably due to the average fiber diameter and mesh porosity. This is verified for the WE2 mesh, which has fibers with smaller diameters and porosity forming a more compact structure that is difficult for water integration. The hydrophilicity of a material is an important parameter to take into account, since it contributes significantly to cell attachment, cell proliferation, and cell migration [4].

### 3.4. Mechanical Properties

**Figure 3:**
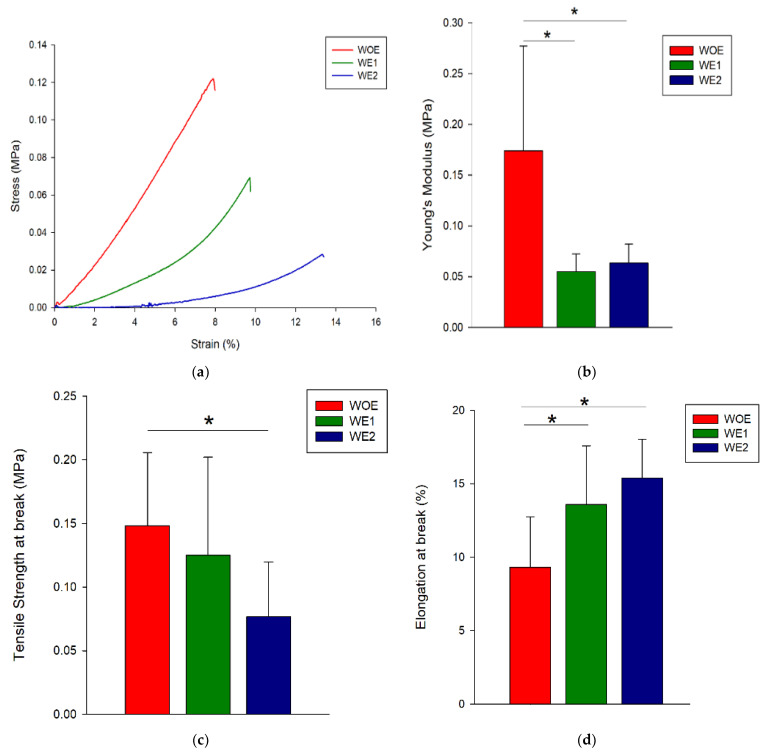
Mechanical behavior of WOE, WE1, and WE2 electrospun meshes in the wet state. (**a**) Stress–strain representative curves, (**b**) Young’s modulus, (**c**) tensile strength at break, (**d**) elongation at break. Statistical significance for *p* ≤ 0.05 (*), n = 15.

The wound dressings are expected to degrade with the new tissue formation and must provide adequate mechanical resistance to support the physiological load [60] and keep its characteristics during the application [60]. The tensile properties of WOE, WE1, and WE2 meshes were evaluated on the wet state since a wet environment is characteristic of wounds. Representative stress–strain curves for each condition are shown in Figure 3a. From those curves, it was possible to obtain Young’s modulus (YM), the tensile strength at break (TSB), and the elongation at break (EB) as shown in Figure 3b–d, respectively. YM measures the solid material stiffness, that is defined by the relationship between stress and strain in the linear elastic regime, i.e., a stiffer material presents higher YM while an elastic material presents lower YM [74]. Regarding the Figure 3b it is possible to observe that by adding the phlorotannins-enriched extract the YM decreases for both conditions (WE1 and WE2) because the structure elasticity increases compared to the control. The amount of extract added does not influence the YM since both samples present the same value of 0.06 ± 0.02 MPa. The TSB for WE1 and WE2 samples decreases when compared with the control meshes which may be due to the un-reacted epoxides of BDDGE that might be attached to the polymers and weakening the interactions between chains increasing the macromolecules mobility and affect directly the mechanical properties [63]. Indeed, the possibility of the hydroxyl groups of phlorotannins to bond with gelatin’s polar groups by hydrogen bonds and chitosan is expected to improve the mechanical properties of electrospun fibers [26,75]. Regarding the TSB and EB, it is possible to observe a contribution of phlorotannins-enriched extract to obtain elastic structures since the structures (WE1 and WE2) support less stress but support more elongation before break compared to the control. This behavior was also observed by Farshi and colleagues [60], in which higher extract content acted as a reinforcing bridge and thus increased the resistance to deformation. Similar results were obtained by Parker and co-authors [43] when comparing poly (vinyl alcohol) PVA hydrogels and blends of PVA and phlorotannins enriched extracts. They observed that hydrogels blended with phlorotannins had decreased Young’s modulus and tensile strength values, and increased values of elongation at the break due to the nature of phlorotannins, which disrupts the crystallites of PVA [43].

It is important to highlight that human skin Young’s modulus is recorded between 2.9-150 MPa, 1-32 MPa for tensile strength, and 17–207% for elongation at break [12]. Overall, gelatin/chitosan loaded with phlorotannins-enriched extract exhibit values generally lower than natural skin, although this behavior can be easily improved by developing hybrid structures combining the fibers developed with synthetic materials to improve the mechanical properties and mimic the native skin properties.

Statistically, there are significant differences between WOE meshes and meshes with phlorotannins-enriched extract for all mechanical tests, with an exception for tensile strength where only WE2 mesh had differences in relation to the WOE meshes. Indeed, the possibility of the hydroxyl groups of phlorotannins to bond with gelatin’s polar groups by hydrogen bonds, and chitosan are expected to improve the mechanical properties of electrospun fibers [26,75].

The results demonstrate that WOE meshes containing gelatin/chitosan had a much higher resistance to stress, likely due to the ionic interaction between gelatin and chitosan, which allows the integrity of electrospun fibers. However, WOE samples are the first to break when compared with the WE1 or WE2 meshes, as a result of chitosan and gelatin having brittle behavior [65].

The extract incorporation apparently changes the electrospun meshes elasticity, as observed by the differences between control samples and those with extract (*p* < 0.05) in Young’s Modulus.

### 3.5. Hydrolytic and Enzymatic Degradation

Electrospun meshes are expected to be biodegradable and absorbable with a proper rate to match the rate of new tissue formation, playing an important role in the regulation of cell proliferation and tissue regeneration [76,77]. Weight loss is a direct measurement to quantify polymer degradation. Based on this, hydrolytic and enzymatic degradation was performed evaluating the weight loss over 28 days (Figure 2B).

According to the hydrolytic results (Figure 2B left)), electrospun WOE meshes present a constant degradation rate over the days. The samples degradation can be correlated with previous parameters tested, like swelling degree and dissolvability. WOE samples had the lowest water uptake, and thus are less susceptible to suffering degradation since the sample area in contact with the degradation medium is lower than the other conditions. On the other hand, WE1 samples had the highest porosity and dissolvability, consequently, the medium penetrates easily into the mesh inducing faster degradation. The WE2 samples present a higher swelling degree due to the hydrophilic character of the extract; however, due to the compact structure (lower fibers diameter) it is difficult for the medium to penetrate into the meshes as observed through the contact angle assay. Regarding the enzymatic degradation (Figure 2B right)), it is noticed that WOE meshes have a higher degradation in the first 15 days, around 80% reaching 82% after 28 days. In fact, lysozyme induces the degradation of chitosan (β-1, 4 N-acetyl-glucosamine groups) and hydrolyzation of gelatin (amino and carboxyl groups) [72]. The WE1 and WE2 meshes show a similar degradation kinetics, keeping the degradation rate constant over the first 15 days and increasing slightly in the last time-points, but not exceeding 40% of degradation. These results suggest that extract compounds have an inhibitory action in this enzyme, as confirmed by the study of Rocasalbas et al. [75]. Polyphenols are known to interact with a variety of proteins via hydrogen bonding and hydrophobic interactions, forming complexes that modulate enzymatic bioactivity [78]. The incorporation of phlorotannins-enriched extract into the fibers might reduce the accessibility of enzyme to the attacking groups of gelatin/chitosan molecules, by reinforcing the bondings between components. According to the results, the produced structures are stable over time therefore can be used for long-term applications as chronic wounds.

### 3.6. In Vitro Phlorotannins-Enriched Extract Release

Among the various electrospinning applications, drug delivery is one of the most promising due to the high loading capacity, high encapsulation efficiency, simultaneous delivery of diverse therapies, ease of operation, and cost-effectiveness [79]. The phlorotannins-enriched extract release from electrospun fibers is outlined in Figure 2C.

As noticed in Figure 2C, both samples comprise two stages of release, with an initial burst release followed by a sustained release for 160 days (5.0 months) of incubation and achieving a cumulative extract release of 98%. The burst release was observed for both meshes containing the extract, after 24 h of the experiment beginning, WE1 mesh releases around 32% of its load, and WE2 mesh releases 35%. This can be likely ascribed to the extract remains in the fibers surface, which is released instantaneously as soon as the membrane is placed in the PBS. The burst release is a result of changes in porosity in polymeric matrices, which allows the extract diffusion out rapidly when the meshes came into contact with the release medium, while a more compact mesh network structure restricts the movement and relaxation of network chains as reported by Park [80]. After that, the extract release rate decreases. The steady phase can occur in one of two ways: erosion or diffusion, since the mesh is constituted by biodegradable polymers. The release of drugs from biodegradable polymers generally is driven by the combination of both mechanisms, which depends on the relative rates of erosion and diffusion [81]. Most biodegradable polymers used for drug delivery are degraded by hydrolysis, since water molecules break the chemical bonds along the polymer chain, the physical integrity of the polymer degrades and allows the drug to be released [81]. Nevertheless, the physical integrity of the meshes was preserved over time, as a result of the strong compounds interactions, as can be seen in Figure 2D. Only narrow changes are visible as compared with the initial state of the structure due to the impossibility of achieving a 100% crosslinking, as reported previously. It is known that gelatin and chitosan interact with each other by hydrogen and ionic bonds, in turn, these compounds also interact with phenolic compounds via hydrogen bonding and ionic interactions with amino groups [35], and these links are reinforced with the addition of BDDGE. In fact, the incorporation of tannic acid (TA), a type of polyphenol, in chitosan films, led to a more rigid and compact matrix, due to TA acting as a crosslinker [82]. Lu et al., also used TA as a crosslinking agent between chitosan and gelatin [26]. Moreover, the fact that the pH of the PBS medium was slightly acidic (pH = 6), to mimic the wound exudate conditions, might have influenced the release of the extract, as reported by Estevez-Areco et al. [83] that verified a smaller amount of polyphenols release in acid conditions that in hydrophilic mediums as a result of reinforcement of the crosslinking process in those conditions. Talón and colleagues also proved that polyphenols release from starch-chitosan films is inhibited when 3% (*v*/*v*) acid acetic was used [82]. According to the possible interactions, in this work, a complete extract release was not possible to achieve.

The extract release mechanism can be characterized by mathematical models, as observed in Table 2. There are several models available to evaluate the release kinetic. Korsmeyer-Peppas showed a significant correlation coefficient with R^2^ = 0.98 and 0.99 for WE2 mesh and WE2, respectively.

A Fickian release was observed in both meshes extract release, since, n values are <0.5 [82], with 0.25 for the mesh loaded with 1 wt% of extract, and 0.35 for the mesh loaded with 2wt% of extract. Those values indicate that the extract release occurs by diffusion, although, other factors associated with drug release must be accounted, such as the hydrophobicity of the drug, the content of loading drug in matrix, and drug distribution [84].

The observed two stages of extract release are an advantage in the therapeutic strategy to treat wounds since burst release confers protection to the wound in the first stage of inflammation, which begins immediately after the tissue injury, while second and steady phase allows the release of the extract in a prolonged manner [85]. This can be an asset for chronic wounds, since their repair requires a longer time, in some cases, months [35].

### 3.7. Antimicrobial Activity

**Figure 4:**
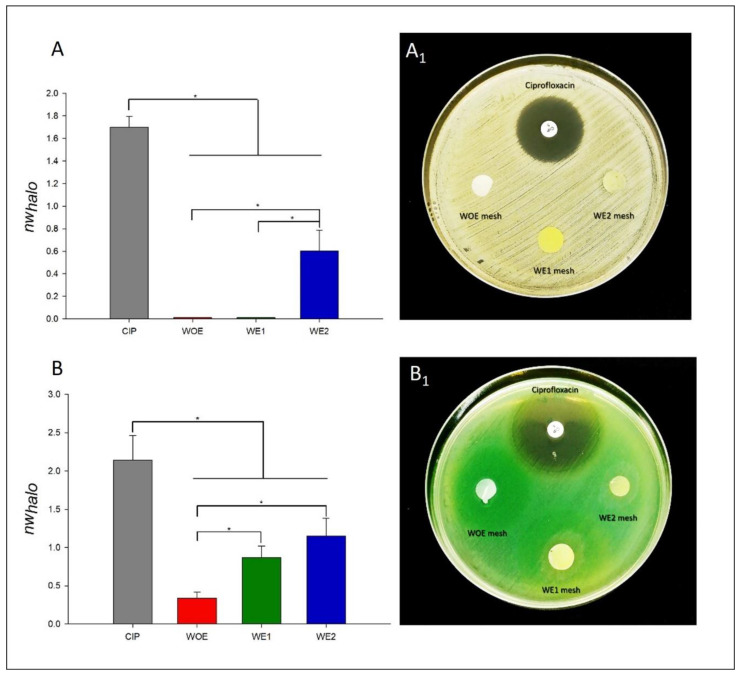
Measurements of the normalized “halo” (*nw_halo_*) for WOE, WE1, and WE2 disc, compared with the control conditions, the antibiotic (ciprofloxacin- CIP) against *Staphylococcus aureus* (**A**) and the photo of the disc assay (**A_1_**). Measurements of the normalized “halo” (*nw_halo_*) for WOE, WE1, and WE2 disc, compared with the control conditions, the antibiotic (ciprofloxacin- CIP) against *Pseudomonas aeruginosa* (**B**) and the photos of the disc assay (**B_1_**). The differences are statistically significant with *p* < 0.05, (*).

The persistence of bacteria in the wound bed, and its increasing resistance to antibiotics, are factors that delay wound healing. *S. aureus* and *P. aeruginosa* are frequently found in wounds, especially in chronic wounds. Data from Europe suggest that 64% of wounds treated in home care are chronic and 24% of these are estimated to persist for 6 months or more, and almost 16% had remained unhealed for a year or more [6]. While *S. aureus* is an opportunistic pathogen usually detected in the top layer of wounds, *P. aeruginosa* is localized in the deepest region of the wound bed [34]. The concern about wound infection is that it is caused by several co-culture of pathogenic bacteria, creating a biofilm and resulting in synergistic effects. Wound healing is delayed because the biofilms are considered a physical obstruction and thus the inflammatory phase is continually extended [86]. In Europe, wound infection had a great contribution to the increased costs to the health systems increasing in average, 11 days to the in-patient hospital stay, with an average cost of €5800 per patient [6]. Thus, it is urgent to develop effective antimicrobial wound dressings. Based on this, antibacterial activity of WOE, WE1, and WE2 meshes against *S. aureus* and *P. aeruginosa* was analyzed and the results are presented in Figure 4—ciprofloxacin was used as control.

Disc diffusion assay produces predominantly quantitative results, which allow us to obtain preliminary information about the antimicrobial activity of electrospun meshes with seaweed extract incorporated. Through Figure 4A,A_1_, the results demonstrated a bacterial inhibition for *S. aureus* for the antibiotic disc (1.701 ± 0.093) as expected, and for WE2 with 0.602 ± 0.182, although, without statistical significance (*p* > 0.05). The conditions WOE and WE1 only present a narrow inhibition, however not sufficient to quantify it. In fact, in literature, many studies have found that Gram-positive bacteria, such *S. aureus* are more resistant to electrospun meshes mechanisms of action, due to the differences in cell walls, in which Gram-positives possess a peptidoglycan layer much thicker overlying the plasma membrane (the target) when compared to Gram-negatives, possibly acting like a protective layer [87,88].

On the other hand, antibacterial activity was observed against *P. aeruginosa* for all conditions (Figure 4B,B_1_), with a *nw_halo_* of 0.338 ± 0.079 for WOE meshes, 0.869 ± 0.151 for WE1 meshes, 1.148 ± 0.237 for WE2 meshes, when compared to control ciprofloxacin with 2.141 ± 0.318 (*p* > 0.05). This possibly is due to the higher hydrophilicity and negative surface charge density (SCD) of Gram-negative bacteria because of the presence of lipopolysaccharide (LPS), that consequently, confer to Gram-negative bacteria a greater affinity to chitosan. Chitosan antimicrobial activity has been reported in several studies, due to the presence of amine groups (NH_2_), making it positively charged in acidic conditions [89] and thus having a higher affinity for the negative charges of Gram-negatives cell wall, leading to a build-up and increased uptake of ions, which then cause intracellular damage [87,88]. In the case of *P. aeruginosa*, the fact that there are no differences between WE1 and WE2 (*p* > 0.05), can be attributed to fibers diameter/porosity, since the WE2 meshes are more compact as a result of their smaller fibers, and the antimicrobial compounds of the extract might be less available. These results can also be correlated with higher crosslink degree since WE2 meshes have a higher amount of extract that acts as crosslinking. Thus, the antimicrobial compounds of the extract might be entrapment into the structure and not released when compared with the WE1 fibers. The obtained phlorotannins-enriched extract already shows antimicrobial activity against both pathogens, with 43.29% ± 2.85 for *S. aureus* and 44.79% ± 8.12 for *P. aeruginosa* [33], and phlorotannins have antimicrobial activity against several described pathogenic bacteria [90,91], being a major asset when applied in electrospun wound dressings, since *S. aureus* and *P. aeruginosa* are responsible for very destructive virulence factors, extending the inflammatory phase, maintaining infection and delaying the wound healing [90]. The virulence factors produced by the *S. aureus* are mainly coagulase, catalase, clumping-factor A, and leucocidines, while *P. aeruginosa* is the main producer of elastase [5]. Those bacterial outputs lead to an imbalance of synthesis and degradation resulting in tissue destruction. Besides, *P. aeruginosa* presents a high resistance to the majority of the antibiotics such as ampicillin, amoxicillin, and others [5]. Regarding the WE1 and WE2 samples, they do not have an antibiotic-like inhibition, but their antimicrobial activity should not be excluded. It is important to note that the antibiotic in question has a great efficacy for a broad spectrum of bacteria and acts by destroying bacterial DNA [92], and the phlorotannins-enriched extract possibly acts by another mechanism, that are not directly comparable. Nevertheless, other factors can influence the result of the diffusion susceptibility test, such as, the antibiotic impregnation and diffusion. Contrary to the commercialized antibiotic, the samples in this study are crosslinked, avoiding a 100% release. Additionally, determining the kinetics of the extract release in agar medium might be difficult as a result of strong hydrogen bonding groups of gelatin (-OH, the-COOH end groups and -NH_2_, and the side groups), chitosan (-OH and the -NH_2_ groups), and phlorotannins hydroxyl groups (-OH), which could result in slow diffusion of the extract from the polymer matrix into the agar medium [93]. However, this methodology is a useful and important tool that helps to choose the most promising electrospun mesh. Thus, those results confirm the potential of electrospun as antimicrobial dressings, however, other quantitative analyses are further required to evaluate the effectiveness of action of the electrospun meshes dopped with the extract.

### 3.8. Biological Behavior

#### 3.8.1. Cytotoxicity

**Figure 5:**
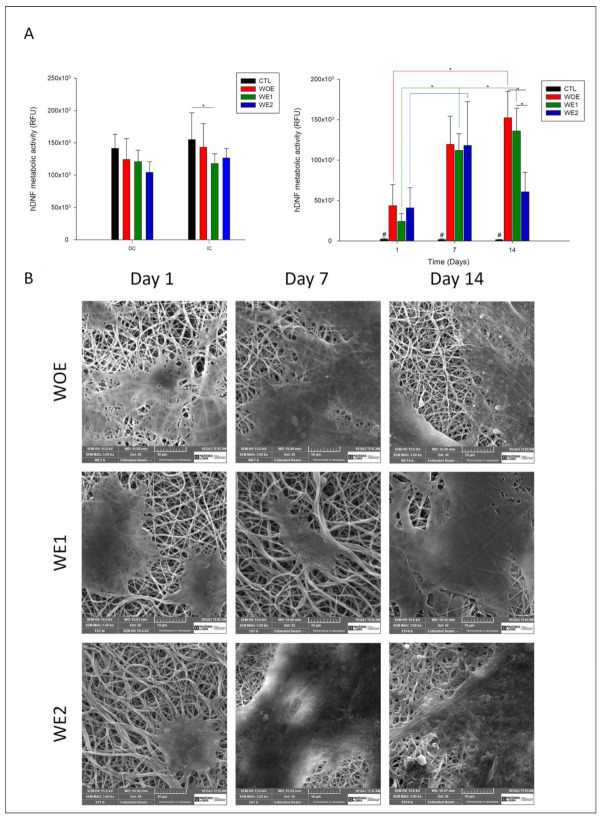
(**A**) On left: Cytotoxicity of electrospun meshes containing phlorotannins-enriched extract (WE1-green bar; WE2-blue bar) and without extract (WOE-red bar) were evaluated by direct (DC) and indirect contact (ID) with a positive control (black bar) of viable cells. Data are expressed as the mean ± SD. Multiple group comparisons were performed using t-test with *p* ≤ 0.05 (*), n = 4. On right: Proliferation of hDNF cells on WOE meshes (red bars), on WE1 meshes (green bars), and WE2 (blue bars), assessed for 1, 7, and 14 days compared with the control meshes (without cells seeded; negative control–black bar). For each time point and multiple groups ANOVA one-way *p* ≤ 0.05 (*) was used, “#” for statistical significance compared with all other samples. (**B**) SEM micrographs representative of hDNF proliferation in different electrospun meshes over 14 days with a magnification of 3000 × (scale bar: 10 µm).

The electrospun meshes cytotoxicity was evaluated both by direct contact (DC) and indirect contact (IC), comparing the WOE meshes with WE1 and WE2 meshes, where the control condition included only viable cells (Figure 5A left).

Through the resazurin assay it was possible to evaluate if electrospun meshes containing phlorotannins-enriched extract induce cytotoxic effect in hDNF cells or not (Figure 5A). The overall results revealed that after 24 h the electrospun meshes did not present toxicity for all conditions in DC and IC assays. Although, in the direct contact, WE2 meshes show statistical differences (*p* < 0.05) when compared with the WOE samples. This can be attributed to extract concentration into the mesh, which can exceed the maximum safe dose, since other authors reported that phlorotannins are safe and can enhance human trachea fibroblast at concentrations of 25 and 50 μg·mL [94]. Other similar studies also revealed that increasing the phlorotannins concentration between 31.25 and 250 μg·mL does not cause adverse effects on human vocal fold fibroblasts [95]. The other conditions, especially WOE meshes (only with gelatin and chitosan) almost reached the same values as the control of viable cells, without statistical differences (*p* > 0.05). This could be explained by the release of uncrosslinked gelatin to the medium [12], and chitosan, due to their similarities with the glycosaminoglycans (GAGs) of the ECM, which are responsible for cell growth regulation, proliferation, cell adhesion, which are features needed in wound repair. Both polymers, gelatin, and chitosan, are non-toxic and biocompatible, which makes them, potential polymers in tissue engineering [29]. The WE1 meshes demonstrated do not affect the viability of hDNF cells, which can be corroborated with the proliferation assays in the next section.

#### 3.8.2. Cell Metabolic Activity and Proliferation

The metabolic activity of the hDNF cells throughout 14 days is presented in Figure 4A) on the right, while in Figure 5B the SEM images are representative of the cell attachment and proliferation for the different electrospun nanofibers at day 1, 7, and 14. According to the results, over 14 days the cells remain metabolically active and increase their activity between time-points, with exception for the WE2 meshes, in which their activity decreases after day 7. The observed results can be attributed to the porosity of the structure, which must allow the passage of nutrients and oxygen. However, the ideal pore size is also associated with the cell type, and for epithelial cells, like fibroblasts, the size of the required fiber varies between 10 and 300 nm, to mimic the native ECM [21], as recorded for the WOE meshes and WE1 meshes. The decrease in fibroblast metabolic activity for WE2 meshes from day 7 can be associated to their smaller fiber diameter when compared to two other conditions, making it a more compact structure, which is difficult for the deep penetration of the cells. Other studies report similar cases. Sisson et al. [96] produced gelatin matrices with different fibers diameters, with small (100 µm ± 40) and bigger diameters (600 µm ± 110), and after two weeks of cultured osteoblastic MG63 cells, meshes with large fiber diameters had an infiltration depth of 50 µm, compared with the 16 µm in fibers with small pores. Other studies claim that chitosan structures with a fiber diameter of 300 nm could increase the proliferation and promote the retention of the chondrocytes than diameters fibers with 1 µm [97] as verified for WOE meshes and WE1. At all time-points the WOE meshes presented higher activity due to gelatin’s RGD peptide sequence (Arg-Gly-Asp) that promotes cell adhesion, migration, and proliferation through the interaction with cell surface receptors [16], as well as chitosan, which is biocompatible, and in several studies shows to have potential wound healing capacity, promoting cell attachment and enhancing wound closure [45]. The observed results are corroborated with the literature, where it has been reported that gelatin/chitosan nanofibers have superior cell attachment and proliferation compared to the nanofibers with the single compounds [66]. Their association with phlorotannins-enriched extract can provide a suitable environment in electrospun meshes for cells to spread and infiltrate, promoting skin regeneration efficiently.

The SEM images show the increase of hDNF cells integration and spread all over the structure through the days, which is demonstrated by the spindle-like shape of fibroblasts phenotype preservation. If at day 1 the cells are only in the meshes surface, at day 7 it is possible to observe the hDNF integration in the electrospun structure. After 14 days, the cells proliferate over the structure, and for the WE2 meshes the cells seem to be only on the surface, which can be correlated to the lower structure porosity and might be also correlated to protein adsorption disorder due to the formation of a water layer that acts as a barrier in this super hydrophilic structure, as previously observed in swelling degree and WCA, as a result of the abundance of polar groups such as amine, hydroxyl, and carboxyl of gelatin, chitosan, and the phlorotannin extract [98].

Thus, the incorporation of the phlorotannin-enriched extract in gelatin/chitosan meshes seems to allow hDNF cells adhesion and proliferation as observed in Park et al. study [43]. The mechanism of how phlorotannins induce cell proliferation is not completely understood, although, according to Guo et al. study, the moieties of polyphenols can also establish chemical bonds to cells and tissue reacting with the nucleophilic groups such as -NH_2_ or -SH and promote cell attachment and proliferation [99]. Another factor can be attributed to the hydroxyl groups of phlorotannins, which seem to increase the hydrophilicity of the structures, and materials with a high hydrophilic profile have greater potential for interacting with biological milieus [66]. Similar to Kim and Wang studies, cell proliferation gradually increases for WE1 meshes and, in fact, phlorotannin extracts have already shown that they do not cause adverse effects on human dermal fibroblasts cells and promote their proliferation [100,101].

An ideal wound dressing must comprise several characteristics covering not only the wound but also providing an adequate environment to stimulate skin regeneration. According to the characterizations performed, the addition of phlorotannins-enriched extract improves mainly the meshes’ properties compared to the electrospun meshes without extract. The most notorious change is related to the mechanical properties, in which the structures containing extract become elastic contrary to the WOE that has a stiffer behavior. The enzymatic degradation kinetics demonstrated constant degradation rate due to the presence of extract that indicates their dual function (antimicrobial and crosslinker). In a general way, there are no significant differences between WE1 and WE2, however, the WE2 in vitro assays demonstrated that the low porosity due to the low fiber diameter makes the cells’ deep integration difficult, presenting a sharp decline of metabolic activity after 7 days in culture. Thus, the WE1 structure may be considered ideal to be used as a wound dressing and as a drug delivery system for long-term applications.

## 4. Conclusions

The main purpose of this work was to explore, for the first time, the addition of phlorotannins-enriched extract into gelatin/chitosan electrospun fibers and develop a multifunctional wound dressing. The structures were produced, characterized, and tested regarding their potential as wound dressing and drug delivery system. Electrospun meshes were dopped with up to 2 wt% of *Undaria pinnatifida* extract concentration, and all conditions were successfully produced, allowing to obtain meshes with well-defined morphology and a random deposition that correctly mimic native skin ECM. The introduction of the phlorotannins-enriched extract seems to reduce the average fiber diameters ranging from 388 ± 82 nm for WOE, 302 ± 83 nm for WE1, to 229 ± 43 nm WE2. Nevertheless, all electrospun meshes conditions possess an ideal porosity (~90%) to act as a wound dressing. The addition of phlorotannins-enriched extract affects the mechanical properties, mainly, the increase of elastic behavior (13.59 ± 3.99 MPa for WE1 and 15.37 ± 2.63 for WE2) as a result of hydroxyl groups of phlorotannins to bond with gelatin’s polar groups by hydrogen bonds, and chitosan. Those interactions also contribute to the stability of the structure over time and in an enzymatic environment, in which degradation rates decrease, compared with the control conditions, fitting long-term applications like chronic wounds. Chronic wounds are the ones posing the highest costs to the health systems and is associated with a long time of healing. The extract release kinetic profile can deliver ~98% of the extract over 160 days, while the electrospun structure maintains its integrity. The antimicrobial results reveal a higher activity against *P. aeruginosa* over *S. aureus* independently of the amount of extract incorporated, being a major asset to combat the infection of depth wounds and avoiding the formation of biofilms. Cytotoxicity assays revealed no toxicity, and proliferation assays showed that fibroblasts were able to attach and proliferate, except for the WE2 samples after day 7. Overall, this study demonstrated the potential of phlorotannins-enriched extract to be an alternative to synthetic antibiotics and the developed electrospun meshes to be used as multifunctional wound dressings.

## Figures and Tables

**Table 1:** Properties of produced electrospun meshes. Mechanical properties correspond to tests performed on the wet state. Statistical significance for *p* ≤ 0.05 using *t*-test, n = 5 (with the exception for mechanical tests n = 15). ^a^ Have statistical significance when compared to control (*p* ≤ 0.05). ^b^ Have statistical significance when compared to 2 wt% of extract (*p* ≤ 0.05).

\	Apparent Density (g·cm^−3^)	Porosity (%)	Average Fiber Diameter (nm)	Swelling Degree (%)	Dissolvability (%)	WVP (g·m^−2^·day^−1^)	Young’s Modulus (MPa)	Tensile Strength at Break (MPa)	Elongation at Break (%)
WOE	0.30 ± 0.09	88. 63 ± 1.81	388 ± 82	405.33 ± 19.11	9.92 ± 0.67	1207.06 ± 14.97	0.174 ± 0.103	0.15 ± 0.06	9.30 ± 44
WE1	0.25 ± 0.03	89.22 ± 1.15 ^b^	302 ± 83 ^a,b^	458.03 ± 41.52 ^a,b^	16.35 ± 0.52 ^a,b^	1220.76 ± 12.06	0.055 ± 0.017 ^a^	0.13 ± 0.08	13.59 ± 3.99 ^a^
WE2	0.31 ± 0.09	85.93 ± 1. 58 ^a^	229 ± 43 ^a^	516.90 ± 39.75 ^a^	9.05 ± 0.69	1201.056 ± 6.00	0.063 ± 0.019 ^a^	0.08 ± 0.04 ^a^	15.37 ± 2.63 ^a^

**Table 2:** Drug release kinetic parameters of electrospun meshes fitted with Korsmeyer-Peppas equation (*K* is the release rate constant; *n* is the release exponent that characterizes the release mechanism).

Sample	*n*	*K*	R^2^
WE1	0.25 ± 0.009	13.03 ± 0.87	0.98
WE2	0.35 ± 0.0095	14.69 ± 0.98	0.99

## Data Availability

Not applicable.

## Supplementary Materials

The following are available online at https://www.mdpi.com/article/10.3390/pharmaceutics13122152/s1, Figure S1: Water contact angle vs time profile of the electrospun meshes.

## References

[1] Vig K., Chaudhari A., Tripathi S., Dixit S., Sahu R., Pillai S., Dennis V., Singh S. (2017). Advances in skin regeneration using tissue engineering. Int. J. Mol. Sci..

[2] Augustine R., Kalarikkal N., Thomas S. (2014). Advancement of wound care from grafts to bioengineered smart skin substitutes. Prog. Biomater..

[3] Wild S., Roglic G., Green A., Sicree R., King H. (2004). Global prevalence of diabetes: Estimates for the year 2000 and projections for 2030. Diabetes Care.

[4] Kim M., Kim G. (2012). Electrospun PCL/phlorotannin nanofibres for tissue engineering: Physical properties and cellular activities. Carbohydr. Polym..

[5] Bessa L.J., Fazii P., Di Giulio M., Cellini L. (2015). Bacterial isolates from infected wounds and their antibiotic susceptibility pattern: Some remarks about wound infection. Int. Wound J..

[6] Lindholm C., Searle R. (2016). Wound management for the 21st century: Combining effectiveness and efficiency. Int. Wound J..

[7] Halstead F.D., Rauf M., Bamford A., Wearn C.M., Bishop J.R.B., Burt R., Fraise A.P., Moiemen N.S., Oppenheim B.A., Webber M.A. (2015). Antimicrobial dressings: Comparison of the ability of a panel of dressings to prevent biofilm formation by key burn wound pathogens. Burns.

[8] Sen C.K. (2019). Human wounds and its burden: An updated compendium of estimates. Adv. Wound Care.

[9] Posnett J., Gottrup F., Lundgren H., Saal G. (2009). The resource impact of wounds on health-care providers in Europe. J. Wound Care.

[10] Dhivya S., Padma V.V., Santhini E. (2015). Wound dressings—A review. BioMedicine.

[11] Wound Care Market—Global Forecast to 2026|MarketsandMarkets. https://www.marketsandmarkets.com/Market-Reports/wound-care-market-371.html.

[12] Dias J.R., Baptista-Silva S., De Oliveira C.M.T., Sousa A., Oliveira A.L., Bártolo P.J., Granja P.L. (2017). In Situ crosslinked electrospun gelatin nanofibers for skin regeneration. Eur. Polym. J..

[13] Dias J.R., dos Santos C., Horta J., Granja P.L., Bártolo P.J. (2017). A new design of an electrospinning apparatus for tissue engineering applications. Int. J. Bioprinting.

[14] Wang F., Hu S., Jia Q., Zhang L. (2020). Advances in electrospinning of natural biomaterials for wound dressing. J. Nanomater..

[15] Kalantari K., Afifi A.M., Jahangirian H., Webster T.J. (2019). Biomedical applications of chitosan electrospun nanofibers as a green polymer—Review. Carbohydr. Polym..

[16] Miguel S.P., Figueira D.R., Simões D., Ribeiro M.P., Coutinho P., Ferreira P., Correia I.J. (2018). Electrospun polymeric nanofibres as wound dressings: A review. Colloids Surf. B Biointerfaces.

[17] Khajavi R., Abbasipour M., Afshari M. (2017). Controlling Nanofiber Morphology by the Electrospinning Process.

[18] Balusamy B., Senthamizhan A., Uyar T., Kny E., Uyar T. (2017). Electrospun nanofibrous materials for wound healing applications. Electrospun Materials for Tissue Engineering and Biomedical Applications: Research, Design and Commercialization.

[19] Willerth S.M., Andrews D.L., Lipson R.H., Nann T. (2018). Electrospun nanofibers for diverse applications. Comprehensive Nanoscience and Nanotechnology.

[20] Thenmozhi S., Dharmaraj N., Kadirvelu K., Kim H.Y. (2017). Electrospun nanofibers: New generation materials for advanced applications. Mater. Sci. Eng. B.

[21] Dias J.R., Granja P.L., Bártolo P.J. (2016). Advances in electrospun skin substitutes. Prog. Mater. Sci..

[22] Kajdič S., Planinšek O., Gašperlin M., Kocbek P. (2019). Electrospun nanofibers for customized drug-delivery systems. J. Drug Deliv. Sci. Technol..

[23] Samimi Gharaie S., Habibi S., Nazockdast H. (2018). Fabrication and characterization of chitosan/gelatin/thermoplastic polyurethane blend nanofibers. J. Text. Fibrous Mater..

[24] Jafari J., Emami S.H., Samadikuchaksaraei A., Bahar M.A., Gorjipour F. (2011). Electrospun chitosan-gelatin nanofiberous scaffold: Fabrication and in vitro evaluation. Biomed. Mater. Eng..

[25] González de Torre I., Ibáñez-Fonseca A., Quintanilla L., Alonso M., Rodríguez-Cabello J.C. (2018). Random and oriented electrospun fibers based on a multicomponent, *in situ* clickable elastin-like recombinamer system for dermal tissue engineering. Acta Biomater..

[26] Lu B., Wang T., Li Z., Dai F., Lv L., Tang F., Yu K., Liu J., Lan G. (2016). Healing of skin wounds with a chitosan-gelatin sponge loaded with tannins and platelet-rich plasma. Int. J. Biol. Macromol..

[27] Talebian A., Mansourian A. (2017). Release of Vancomycin from electrospun gelatin/chitosan nanofibers. Mater. Today Proc..

[28] Kenawy E., Abdel-Hay F.I., El-Newehy M.H., Wnek G.E., Linkov I., Steevens J. (2009). Processing of Polymer Nanofibers through Electrospinning as Drug Delivery Systems. Nanomaterials: Risks and Benefits.

[29] Jalaja K., Naskar D., Kundu S.C., James N.R. (2016). Potential of electrospun core-shell structured gelatin-chitosan nanofibers for biomedical applications. Carbohydr. Polym..

[30] Ige O.O., Umoru L.E., Aribo S. (2012). Natural Products: A minefield of biomaterials. Int. Sch. Res. Netw..

[31] Li Y.-X., Wijesekara I., Li Y., Kim S.-K. (2011). Phlorotannins as bioactive agents from brown algae. Process Biochem..

[32] Lopes G., Sousa C., Silva L.R., Pinto E., Andrade P.B., Bernardo J., Mouga T., Valentão P. (2012). Can phlorotannins purified extracts constitute a novel pharmacological alternative for microbial infections with associated inflammatory conditions?. PLoS ONE.

[33] Ferreira C.A.M., Félix R., Félix C., Januário A.P., Alves N., Novais S.C., Dias J.R., Lemos M.F.L. (2021). A Biorefinery approach to the biomass of the seaweed *Undaria pinnatifida* (Harvey Suringar, 1873): Obtaining phlorotannins-enriched extracts for wound healing. Biomolecules.

[34] Serra R., Grande R., Butrico L., Rossi A., Settimio U.F., Caroleo B., Amato B., Gallelli L., De Franciscis S. (2015). Chronic wound infections: The role of *Pseudomonas aeruginosa* and *Staphylococcus aureus*. Expert Rev. Anti. Infect. Ther..

[35] Moeini A., Pedram P., Makvandi P., Malinconico M., Gomez d’Ayala G. (2020). Wound healing and antimicrobial effect of active secondary metabolites in chitosan-based wound dressings: A review. Carbohydr. Polym..

[36] Ford L., Stratakos A.C., Theodoridou K., Dick J.T.A., Sheldrake G.N., Linton M., Corcionivoschi N., Walsh P.J. (2020). Polyphenols from brown seaweeds as a potential antimicrobial agent in animal feeds. ACS Omega.

[37] Kurahashi T., Fujii J. (2015). Roles of antioxidative enzymes in wound healing. J. Dev. Biol..

[38] Félix R., Valentão P., Andrade P.B., Félix C., Novais S.C., Lemos M.F.L. (2020). Evaluating the in vitro potential of natural extracts to protect lipids from oxidative damage. Antioxidants.

[39] Shavandi A., Bekhit A.E.D.A., Saeedi P., Izadifar Z., Bekhit A.A., Khademhosseini A. (2018). Polyphenol uses in biomaterials engineering. Biomaterials.

[40] Gao X., Xu Z., Liu G., Wu J. (2021). Polyphenols as a versatile component in tissue engineering. Acta Biomater..

[41] Guimarães I., Baptista-Silva S., Pintado M., Oliveira A. (2021). Polyphenols: A promising avenue in therapeutic solutions for wound care. Appl. Sci..

[42] Yeo M., Jung W.-K., Kim G. (2012). Fabrication, characterisation and biological activity of phlorotannin-conjugated PCL/β-TCP composite scaffolds for bone tissue regeneration. J. Mater. Chem..

[43] Park H.-H., Ko S.-C., Oh G.-W., Heo S.-J., Kang D.-H., Bae S.-Y., Jung W.-K. (2018). Fabrication and characterization of phlorotannins/poly (vinyl alcohol) hydrogel for wound healing application. J. Biomater. Sci. Polym. Ed..

[44] Kuntzler S.G., Costa J.A.V., de Morais M.G. (2018). Development of electrospun nanofibers containing chitosan/PEO blend and phenolic compounds with antibacterial activity. Int. J. Biol. Macromol..

[45] Pezeshki-Modaress M., Zandi M., Rajabi S. (2018). Tailoring the gelatin/chitosan electrospun scaffold for application in skin tissue engineering: An in vitro study. Prog. Biomater..

[46] Mohammadzadehmoghadam S., Dong Y. (2019). Fabrication and characterization of electrospun silk fibroin/gelatin scaffolds crosslinked with glutaraldehyde vapor. Front. Mater..

[47] The American Society for Testing and Materials (1995). Annual Book of ASTM Standards: ASTM E96-95: Standard Test Methods for Water Vapor Transmission of Materials.

[48] Tallian C., Tegl G., Quadlbauer L., Vielnascher R., Weinberger S., Cremers R., Pellis A., Salari J.W.O., Guebitz G.M. (2019). Lysozyme-responsive spray-dried chitosan particles for early detection of wound infection. ACS Appl. Bio Mater..

[49] Lončarević A., Ivanković M., Rogina A. (2017). Lysozyme-induced degradation of chitosan: The characterisation of degraded chitosan scaffolds. J. Tissue Repair Regen..

[50] Mak Y.W., Leung W.W.F. (2019). Crosslinking of genipin and autoclaving in chitosan-based nanofibrous scaffolds: Structural and physiochemical properties. J. Mater. Sci..

[51] Percival S.L., McCarty S., Hunt J.A., Woods E.J. (2014). The effects of pH on wound healing, biofilms, and antimicrobial efficacy. Wound Repair Regen..

[52] Ramteke K.H., Dighe P., Kharat A.R., Patil S.V. (2014). Mathematical Models of Drug Dissolution: A Review. Sch. Acad. J. Pharm..

[53] Kalani M.M., Nourmohammadi J., Negahdari B., Rahimi A., Sell S.A. (2019). Electrospun core-sheath poly(vinyl alcohol)/silk fibroin nanofibers with Rosuvastatin release functionality for enhancing osteogenesis of human adipose-derived stem cells. Mater. Sci. Eng. C.

[54] Clinical and Laboratory Standards Institute (CLSI) (2014). CLSI Supplement M100S: Performance Standards for Antimicrobial Susceptibility Testing.

[55] Martí M., Frígols B., Serrano-Aroca A. (2018). Antimicrobial characterization of advanced materials for bioengineering applications. J. Vis. Exp..

[56] International Organization for Standardization (2009). “UNI EN ISO 10993-5: 2009” Biological Evaluation of Medical Devices–Part 5: In Vitro Cytotoxicity Testing.

[57] Dias J.R., Baptista-Silva S., Sousa A., Oliveira A.L., Bártolo P.J., Granja P.L. (2018). Biomechanical performance of hybrid electrospun structures for skin regeneration. Mater. Sci. Eng. C.

[58] Sandri G., Rossi S., Bonferoni M.C., Caramella C., Ferrari F., Boateng J. (2020). Electrospinning Technologies in Wound Dressing Applications. Therapeutic Dressings and Wound Healing Applications.

[59] Goh Y.F., Shakir I., Hussain R. (2013). Electrospun fibers for tissue engineering, drug delivery, and wound dressing. J. Mater. Sci..

[60] Farshi Azhar F., Olad A., Salehi R. (2014). Fabrication and characterization of chitosan–gelatin/nanohydroxyapatite–polyaniline composite with potential application in tissue engineering scaffolds. Des. Monomers Polym..

[61] Jridi M., Hajji S., Ayed H.B., Lassoued I., Mbarek A., Kammoun M., Souissi N., Nasri M. (2014). Physical, structural, antioxidant and antimicrobial properties of gelatin–chitosan composite edible films. Int. J. Biol. Macromol..

[62] Nieto-Suárez M., López-Quintela M.A., Lazzari M. (2016). Preparation and characterization of crosslinked chitosan/gelatin scaffolds by ice segregation induced self-assembly. Carbohydr. Polym..

[63] Martucci J.F., Espinosa J.P., Ruseckaite R.A. (2015). Physicochemical properties of films based on bovine gelatin cross-linked with 1,4-Butanediol Diglycidyl Ether. Food Bioprocess Technol..

[64] Amiri N., Rozbeh Z., Afrough T., Sajadi Tabassi S.A., Moradi A., Movaffagh J. (2018). Optimization of chitosan-gelatin nanofibers production: Investigating the effect of solution properties and working parameters on fibers diameter. Bionanoscience.

[65] Noorani B., Tabandeh F., Yazdian F., Soheili Z.-S., Shakibaie M., Rahmani S. (2018). Thin natural gelatin/chitosan nanofibrous scaffolds for retinal pigment epithelium cells. Int. J. Polym. Mater. Polym. Biomater..

[66] Bazmandeh A.Z., Mirzaei E., Fadaie M., Shirian S., Ghasemi Y. (2020). Dual spinneret electrospun nanofibrous/gel structure of chitosan-gelatin/chitosan-hyaluronic acid as a wound dressing: in vitro and *in vivo* studies. Int. J. Biol. Macromol..

[67] Naseri N., Algan C., Jacobs V., John M., Oksman K., Mathew A.P. (2014). Electrospun chitosan-based nanocomposite mats reinforced with chitin nanocrystals for wound dressing. Carbohydr. Polym..

[68] Letha S.S., Kumar A.S., Nisha U., Rosemary M.J. (2021). Electrospun polyurethane-gelatin artificial skin scaffold for wound healing. J. Text. Inst..

[69] Roy S., Zhai L., Chan Kim H., Hoa Pham D., Alrobei H., Kim J. (2021). Tannic-acid-cross-linked and TiO 2-nanoparticle reinforced chitosan-based nanocomposite film. Polymers.

[70] Gu S.Y., Wang Z.M., Ren J., Zhang C.Y. (2009). Electrospinning of gelatin and gelatin/poly(l-lactide) blend and its characteristics for wound dressing. Mater. Sci. Eng. C.

[71] Franco R.A., Nguyen T.H., Lee B.-T. (2011). Preparation and characterization of electrospun PCL/PLGA membranes and chitosan/gelatin hydrogels for skin bioengineering applications. J. Mater. Sci. Mater. Med..

[72] Kim E.H., Lim S., Kim E., Jeon I.O., Choi Y.S. (2018). Preparation of *in situ* injectable chitosan/gelatin hydrogel using an acid-tolerant tyrosinase. Biotechnol. Bioprocess Eng..

[73] Morgado P.I., Aguiar-Ricardo A., Correia I.J. (2015). Asymmetric membranes as ideal wound dressings: An overview on production methods, structure, properties and performance relationship. J. Memb. Sci..

[74] Wang J.C. (1984). Young’s modulus of porous materials—Part 1 Theoretical derivation of modulus-porosity correlation. J. Mater. Sci..

[75] Rocasalbas G., Francesko A., Touriño S., Fernández-Francos X., Guebitz G.M., Tzanov T. (2013). Laccase-assisted formation of bioactive chitosan/gelatin hydrogel stabilized with plant polyphenols. Carbohydr. Polym..

[76] Yang C., Xu L., Zhou Y., Zhang X., Huang X., Wang M., Han Y., Zhai M., Wei S., Li J. (2010). A green fabrication approach of gelatin/CM-chitosan hybrid hydrogel for wound healing. Carbohydr. Polym..

[77] Zheng J.P., Wang C.Z., Wang X.X., Wang H.Y., Zhuang H., De Yao K. (2007). Preparation of biomimetic three-dimensional gelatin/montmorillonite–chitosan scaffold for tissue engineering. React. Funct. Polym..

[78] Kellogg J., Grace M.H., Lila M.A. (2014). Phlorotannins from alaskan seaweed inhibit carbolytic enzyme activity. Mar. Drugs.

[79] Hu X., Liu S., Zhou G., Huang Y., Xie Z., Jing X. (2014). Electrospinning of polymeric nanofibers for drug delivery applications. J. Control. Release.

[80] Hezaveh H., Muhamad I.I. (2013). Controlled drug release via minimization of burst release in pH-response kappa-carrageenan/polyvinyl alcohol hydrogels. Chem. Eng. Res. Des..

[81] Karuppuswamy P., Reddy Venugopal J., Navaneethan B., Luwang Laiva A., Ramakrishna S. (2015). Polycaprolactone nanofibers for the controlled release of tetracycline hydrochloride. Mater. Lett..

[82] Talón E., Trifkovic K.T., Vargas M., Chiralt A., González-Martínez C. (2017). Release of polyphenols from starch-chitosan based films containing thyme extract. Carbohydr. Polym..

[83] Estevez-Areco S., Guz L., Candal R., Goyanes S. (2018). Release kinetics of rosemary (*Rosmarinus officinalis*) polyphenols from polyvinyl alcohol (PVA) electrospun nanofibers in several food simulants. Food Packag. Shelf Life.

[84] Shao S., Li L., Yang G., Li J., Luo C., Gong T., Zhou S. (2011). Controlled green tea polyphenols release from electrospun PCL/MWCNTs composite nanofibers. Int. J. Pharm..

[85] Helary C., Abed A., Mosser G., Louedec L., Letourneur D., Coradin T., Giraud-Guille M.M., Meddahi-Pellé A. (2015). Evaluation of dense collagen matrices as medicated wound dressing for the treatment of cutaneous chronic wounds. Biomater. Sci..

[86] Negut I., Grumezescu V., Grumezescu A. (2018). Treatment strategies for infected wounds. Molecules.

[87] Arkoun M., Daigle F., Heuzey M.C., Ajji A. (2017). Mechanism of action of electrospun chitosan-based nanofibers against meat spoilage and pathogenic bacteria. Molecules.

[88] Slavin Y.N., Asnis J., Häfeli U.O., Bach H. (2017). Metal nanoparticles: Understanding the mechanisms behind antibacterial activity. J. Nanobiotechnol..

[89] Nada A.A., El Aref A.T., Sharaf S.S. (2019). The synthesis and characterization of zinc-containing electrospun chitosan/gelatin derivatives with antibacterial properties. Int. J. Biol. Macromol..

[90] Surendhiran D., Cui H., Lin L. (2019). Encapsulation of phlorotannin in Alginate/PEO blended nanofibers to preserve chicken meat from *Salmonella* contaminations. Food Packag. Shelf Life.

[91] Eom S.-H., Kim Y.-M., Kim S.-K. (2012). Antimicrobial effect of phlorotannins from marine brown algae. Food Chem. Toxicol..

[92] Knoll K.E., Lindeque Z., Adeniji A.A., Oosthuizen C.B., Lall N., Loots D.T. (2021). Elucidating the antimycobacterial mechanism of action of ciprofloxacin using metabolomics. Microorganisms.

[93] Hosseini S.F., Rezaei M., Zandi M., Farahmandghavi F. (2016). Development of bioactive fish gelatin/chitosan nanoparticles composite films with antimicrobial properties. Food Chem..

[94] Lee H.S., Jeong M., Ko S., Heo S., Kang H.W., Kim S.W., Hwang C.W., Lee K.D., Oak C., Jung M.J. (2020). Fabrication and biological activity of polycaprolactone/phlorotannin endotracheal tube to prevent tracheal stenosis: An in vitro and in vivo study. J. Biomed. Mater. Res. Part. B Appl. Biomater..

[95] Kim T.H., Lee H.S., Oh S.J., Hwang C.W., Jung W.K. (2020). Phlorotannins ameliorate extracellular matrix production in human vocal fold fibroblasts and prevent vocal fold fibrosis via aerosol inhalation in a laser-induced fibrosis model. J. Tissue Eng. Regen. Med..

[96] Sisson K., Zhang C., Farach-Carson M.C., Chase D.B., Rabolt J.F. (2010). Fiber diameters control osteoblastic cell migration and differentiation in electrospun gelatin. J. Biomed. Mater. Res. Part. A.

[97] Ameer J.M., PR A.K., Kasoju N. (2019). Strategies to tune electrospun scaffold porosity for effective cell response in tissue engineering. J. Funct. Biomater..

[98] Wei J., Igarashi T., Okumori N., Igarashi T., Maetani T., Liu B., Yoshinari M. (2009). Influence of surface wettability on competitive protein adsorption and initial attachment of osteoblasts. Biomed. Mater..

[99] Guo J., Sun W., Kim J.P., Lu X., Li Q., Lin M., Mrowczynski O., Rizk E.B., Cheng J., Qian G. (2018). Development of tannin-inspired antimicrobial bioadhesives. Acta Biomater..

[100] Kim M.M., Van Ta Q., Mendis E., Rajapakse N., Jung W.K., Byun H.G., Jeon Y.J., Kim S.K. (2006). Phlorotannins in *Ecklonia cava* extract inhibit matrix metalloproteinase activity. Life Sci..

[101] Wang L., Kim H.S., Oh J.Y., Je J.G., Jeon Y.J., Ryu B.M. (2020). Protective effect of diphlorethohydroxycarmalol isolated from Ishige okamurae against UVB-induced damage in vitro in human dermal fibroblasts and in vivo in zebrafish. Food Chem. Toxicol..

