# Supplementary material for: Multifunctional Gelatin/Chitosan Electrospun Wound Dressing Dopped with Undaria pinnatifida Phlorotannin-Enriched Extract for Skin Regeneration
Source: Pharmaceutics. 2021 Dec 14;13(12):2152. doi: 10.3390/pharmaceutics13122152 (PMC8704818; doi:10.3390/pharmaceutics13122152)
Supplement: Supplementary file 1 [file pharmaceutics-13-02152-s001.zip › pharmaceutics-1472210- final Supplementary Materials.pdf]

*pharmaceutics*

# Supplementary Materials: Multifunctional Gelatin/Chitosan Electrospun Wound Dressing Dopped with *Undaria pinnatifida* Phlorotannin-Enriched Extract for Skin Regeneration

**Carolina A. M. Ferreira, Adriana P. Januário, Rafael Félix, Nuno Alves, Marco F. L. Lemos and Juliana R. Dias**

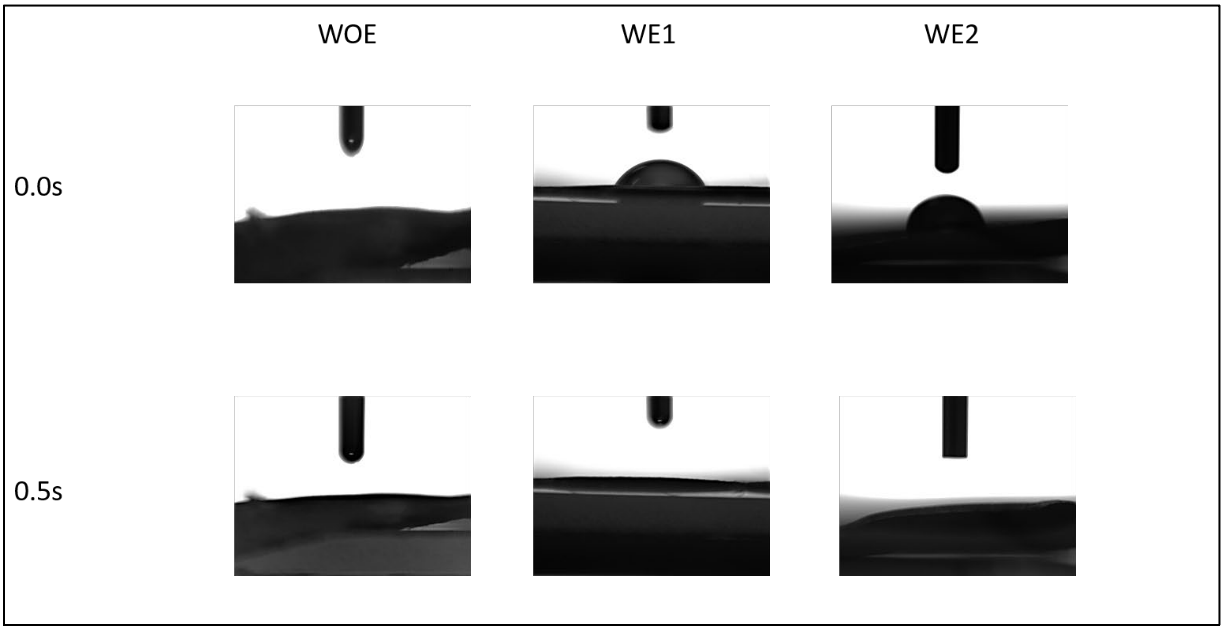


**Figure S1.** Water contact angle vs time profile of the electrospun meshes.
